# Age, Frailty, and Comorbidity as Predictors of Mortality and Failure to Rescue After Gastrointestinal Cancer Surgery: A National Retrospective Cohort Study

**DOI:** 10.1002/wjs.70268

**Published:** 2026-02-14

**Authors:** Cameron I. Wells, Chris Varghese, Greg O'Grady, Ian P. Bissett

**Affiliations:** ^1^ Department of Surgery The University of Auckland Auckland New Zealand; ^2^ Mayo Clinic Rochester Minnesota USA; ^3^ Department of Surgery Auckland City Hospital Auckland New Zealand

**Keywords:** aged, comorbidity, failure to rescue, frailty, gastrointestinal neoplasms

## Abstract

**Background:**

Many older adults undergo gastrointestinal (GI) cancer surgery, yet the relative impacts of advanced age, comorbidity, and frailty on postoperative mortality and failure to rescue (FTR) remain unclear. We aimed to compare the impacts of these factors on postoperative outcomes in a large population‐based cohort.

**Method:**

We conducted a national retrospective linked database study of patients aged ≥ 65 years who underwent resection for GI or hepatobiliary cancer in Aotearoa New Zealand between 2005 and 2020. Age, comorbidity (C3 Comorbidity Score), and frailty (Hospital Frailty Risk Score) were examined as independent predictors of 90‐day mortality, complications, and FTR (death following a complication). Logistic regression models were adjusted for demographic and clinical covariates.

**Results:**

Among 21,729 patients (mean age 75.8 ± 6.8 years), 49.3% experienced one or more complications and 6.1% died within 90 days (FTR rate of 12.3%). In adjusted models, each additional 5 years of age increased 90‐day mortality odds by 40% (adjusted odds ratio [aOR] 1.40 and 95% CI 1.34–1.46). Although higher comorbidity (C3 > 3) and frailty (Hospital Frailty Risk Score > 15) also independently raised mortality risk (aOR 1.98 and 2.04, respectively), age exerted the largest effect on mortality and FTR.

**Conclusion:**

Chronological age, comorbidity, and frailty each predict worse outcomes following GI cancer surgery, but advanced age remains the dominant driver of 90‐day mortality and FTR. These findings underscore the need for risk stratification, shared decision‐making, and preoperative optimization as wells as intense surveillance for complications and early definitive care of older adults undergoing major oncologic operations.

## 1 Background

Populations worldwide are aging, leading to an increasing cancer incidence amongst older adults [1, 2]. Gastrointestinal (GI) malignancies are expected to contribute to a large proportion of the anticipated increase in cancer incidence over the next 50 years [1], many of whom will require surgery. Notably, the rate of aging among surgical patients is outpacing that of the general population [3], emphasizing the pressing need to better understand and mitigate postoperative risks in older adults undergoing major cancer operations.

Beyond chronological age alone, older patients frequently face overlapping syndromes that impact surgical outcomes, notably comorbidity and frailty. Comorbidity is common amongst older patients with cancer, reflecting the increasing burden of chronic disease amongst an aging population [4]. Frailty is a geriatric syndrome defined as a state of vulnerability to poor resolution of homeostasis after a stressor event and is the result of cumulative decline in many physiological systems during a lifetime [5]. Collectively, these factors contribute to higher complication rates and greater risks of mortality. “Failure to rescue” (FTR), defined as the rate of mortality amongst patients with complications [6], has previously been shown to be a major driver of poor outcomes for elderly patients.

However, despite recognition that age, comorbidity, and frailty each predict worse surgical outcomes, limited population‐level data directly compare the relative impact of these three related factors, especially in the context of postoperative complications and FTR. Such evidence is vital for guiding patient selection, risk stratification, perioperative planning, and identifying patients requiring additional postoperative vigilance for complications and their impact.

Therefore, in this national population‐based cohort study from 2005 to 2020, we aimed to (a) determine the independent association of age, comorbidity, and frailty with 90‐day mortality, complications, and FTR following gastrointestinal and hepatobiliary cancer surgery and (b) compare the relative impact of each factor to better guide clinical decision‐making and perioperative planning for high‐risk patients.

## 2 Methods

We conducted a retrospective population‐based cohort study of patients undergoing resection for gastrointestinal and hepatobiliary cancers in Aotearoa New Zealand from 2005–2020, reported according to the relevant EQUATOR network guidelines [7, 8]. Ethical approval was obtained from the Auckland Health Research Ethics Committee (AH23701), which granted a waiver of individual informed consent for analysis of de‐identified administrative data.

### 2.1 Data Sources

Linked patient data were obtained from the New Zealand Ministry of Health National Minimum Dataset (NMDS) and New Zealand Cancer Registry (NZCR) [9] as previously described [10, 11]. Relevant International Classification of Diseases (ICD) version 10‐AM procedure and diagnosis codes were obtained from all publicly and privately funded hospitalizations in Aotearoa New Zealand. Mortality data were sourced from the National Health Index (NHI) dataset, linked to the New Zealand Mortality Data Collection.

### 2.2 Inclusion and Exclusion Criteria

We included patients aged ≥ 65 years undergoing major gastrointestinal or hepatopancreatobiliary cancer surgery between January 1 2005 and December 31 2020. For inclusion, patients were required to have a new diagnosis of esophagogastric, small intestinal, colorectal, or hepatopancreatobiliary cancer registered in the NZCR within 1 year preoperatively or 2 months following a surgery corresponding to that cancer type. For patients with multiple eligible procedures or cancers during the study period, only the first was considered for analysis. We excluded patients with a cancer diagnosis based on a death certificate only. Patients with appendiceal or gallbladder cancers were also excluded as these pathologies are often initially treated with appendicectomy or cholecystectomy alone (with relatively little associated morbidity) but also frequently involve secondary staged procedures depending on initial histology results, which could not be accurately distinguished in administrative data from unplanned reoperations for postoperative complications.

### 2.3 Variables

The main explanatory variables of interest were chronological age, comorbidity, and frailty:

1. *Chronological age* was measured in years, defined as age at the index hospital admission for surgery.
2. *Comorbidity* was measured using the C3 score [12], a comorbidity measure derived from International Classification of Disease (ICD) 10‐AM codes for 42 conditions (Supporting Information S1: Table 1). This index has been derived and validated in the Aotearoa New Zealand setting [13, 14], with superior predictive performance for mortality amongst cancer patients compared to the Charlson and National Cancer Institute Indices [12].
3. *Frailty* was measured using the Hospital Frailty Risk Score (HFRS) [15], which assigns a point value to 109 frailty‐related ICD‐10 codes (Supporting Information S1: Table 2), including recognized functional deficits, comorbid illnesses, falls, gait abnormalities, and vitamin D deficiency. This measure has previously has been shown to identify those at risk of poor outcomes in older people with medical illness [16] and has superior predictive value for adverse surgical outcomes compared to other commonly used frailty metrics [17].

Both the C3 Comorbidity Score and HRFS were calculated using all ICD codes from all public hospital admissions in New Zealand, during a look‐back period of 5 years prior to the date of surgery. Where possible, we analyzed age, comorbidity, and frailty as continuous variables; however for ease of interpretation of results, in some analyses, these were grouped into categories. For age, this was done using 5‐year blocks, for C3, this was done using discrete groups of “0”, “0–1”, “1–2”, “2–3”, and “≥ 3”, similar to Gurney et al. [16, 17], and for HFRS, we used the cut‐offs of low (< 5), intermediate [5, 6, 7, 8, 9, 10, 11, 12, 13, 14, 15], and high (> 15) risk as proposed by Gilbert et al. [15].

Other variables included sex (defined as male or female), self‐reported ethnicity (priority coded as Māori, Pacific People, Asian, and European/Other), socioeconomic deprivation (measured using the area‐level NZDep2013 index), cancer location, and stage of disease (defined using the Surveillance Epidemiology and End Results database coding). Due to the strong correlation between American Society of Anesthesiologists (ASA) Physical Status Classification and comorbidity, and that ASA scores were missing for 10.7% of the cohort, we elected not to analyze this variable, aiming to avoid bias from multicollinearity and missing data.

### 2.4 Outcomes

The primary outcomes of interest were 90‐day mortality, 90‐day postoperative complications, and FTR, defined as 90‐day mortality following a complication within 90 days of surgery [11]. We included 19 complications; reoperation, endoscopic reintervention, radiological reintervention, blood transfusion, AKI/renal failure, cardiac arrest, arrhythmia, myocardial infarction, sepsis, shock, DVT/PE, pneumonia, respiratory failure, stroke, delirium, surgical site infection, GI bleeding, hemorrhage, and death, defined using ICD‐10 codes, consistent with our previous work [6, 11, 18]. Reoperation, radiological reintervention, and endoscopic reintervention were defined using specific ICD‐10 codes for each cancer type [11]. Secondary outcomes were failure to rescue after each complication (defined as the proportion of patients who died within 90 days of surgery following a given complication), 1‐year and 2‐year mortality, intensive care unit (ICU) admission during the index hospital admission, length of index hospital stay, and unplanned readmission to any public hospital in New Zealand within 90 days of surgery. FTR was also stratified into FTR‐Surgical and FTR‐Nonoperative as previously described [19, 20]. We primarily analyzed 90‐day outcomes as it is well recognized that this timeframe more accurately reflect the impacts of major cancer surgery, particularly for older and frail patients [19, 20].

### 2.5 Missing Data

Data were missing for cancer extent (*n* = 2,580, 11.9%), NZDep13 (*n* = 5, 0.0%), and ethnicity (*n* = 7, 0.0%). There were no meaningful differences in demographic or operative factors or outcomes between patients with and without missing data; therefore, these were assumed to be missing at random. Multiple imputation was used in multivariate analyses to account for missing data.

### 2.6 Statistical Analysis

R (Version 4.4.3, R Foundation for Statistical Computing, Vienna, Austria) was used for all analyses. Categorical variables were compared using chi‐squared tests, and continuous variables were compared using t‐tests or Mann–Whitney U tests as appropriate.

Multivariable logistic regression models were constructed for each outcome (mortality, complications, and FTR), using multiple imputation to address missing data. The aregImpute function from the *Hmisc* package was used to generate 10 multiple imputations using additive regression, bootstrapping, and predictive mean matching. The imputation model included all variables used in the final analysis model. With adjustment for clinically relevant confounders: age, sex, ethnicity, socioeconomic deprivation, admission acuity, cancer type, cancer stage, operation year, C3 Comorbidity Score, and Hospital Frailty Risk Score. Logistic regression models were then fitted separately for each imputed dataset and pooled using Rubin's rules. As Gompertz's law of human mortality has previously been shown to apply to postoperative death [21], age was modeled as a linear term on the log‐odds scale, reflecting an exponential increase in mortality risk with age. To allow for nonlinear associations, the C3 Comorbidity Score and the Hospital Frailty Risk Score were modeled using restricted cubic splines with four knots to allow for non‐linear associations. The number of knots was selected a priori to balance model flexibility with interpretability. Results are reported as adjusted odds ratios (aOR) with 95% confidence intervals (95% CIs). A *p* value of less than 0.05 was considered statistically significant. Subgroup analyses were also undertaken with the cohort stratified into patients with colorectal cancer versus all other cancer groups.

As a sensitivity analysis, we also constructed logistic regression models where age, comorbidity, and frailty were modeled as categorical variables using the groupings described above. To ensure that our results were not potentially biased by patients with advanced disease undergoing palliative surgery, we performed a further sensitivity analysis excluding patients undergoing emergency surgery and with documented distant metastatic disease.

## 3 Results

### 3.1 Cohort Characteristics

**FIGURE 1:**
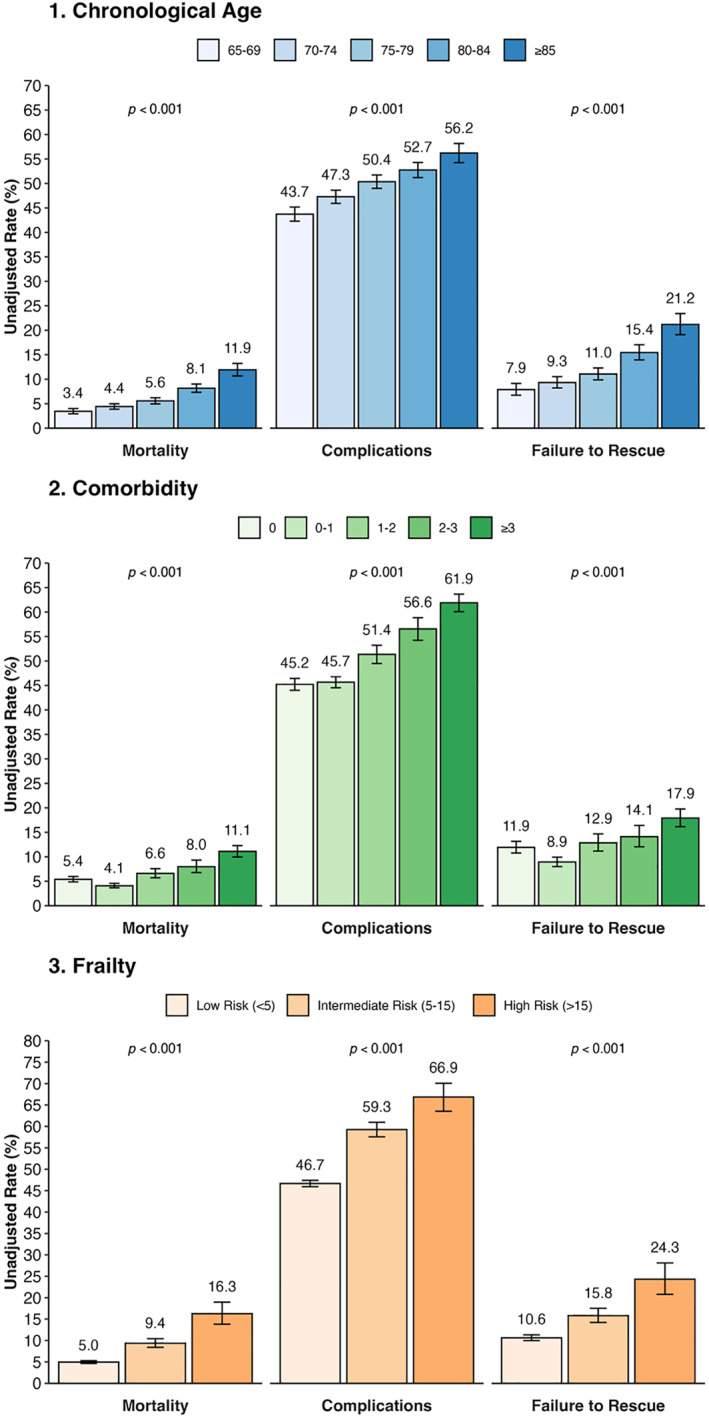
Univariate association of chronological age, comorbidity, and frailty with postoperative mortality, complications, and failure to rescue.

Of a total 31,199 patients undergoing gastrointestinal or hepatobiliary cancer resection during the study period, we included 21,729 individuals aged ≥ 65 years (Supporting Information S1: Figure 1). Patient demographics are detailed in Table 1. The mean age was 75.8 ± 6.8 years (median 75 and IQR 70–81) and 11.7% (*n* = 2535) were ≥ 85 years. Just under half of the cohort were female (47.7%), most had colorectal cancer (89.6%) and 5.0% were indigenous Māori. Proportions of Māori declined amongst older age groups (Table 1). Comorbidity burden increased with age, the mean C3 score increased from 0.9 ± 1.5 in the youngest group (65–69 years) to 1.5 ± 1.9 in those ≥ 85 years (*p* < 0.001). Similarly, the proportion of patients at intermediate or high risk of frailty markedly increased with age (1.8 ± 3.7 vs. 4.7 ± 6.8 and *p* < 0.001).

**TABLE 1:** Demographic and clinical data for the included cohort, stratified by chronological age.

		Age (years)						
Variable		65–69	70–74	75–79	80–84	≥ 85	Total	*p*
Total *N* (%)		4590 (21.1%)	5311 (24.4%)	5230 (24.1%)	4063 (18.7%)	2535 (11.7%)	21,729	
Sex	Female	1882 (41.0%)	2328 (43.8%)	2451 (46.9%)	2166 (53.3%)	1527 (60.2%)	10,354 (47.7%)	< 0.001
	Male	2708 (59.0%)	2983 (56.2%)	2779 (53.1%)	1897 (46.7%)	1008 (39.8%)	11,375 (52.3%)	
Age	Mean (SD)	67.1 (1.4)	72.1 (1.4)	76.9 (1.4)	81.9 (1.4)	87.7 (2.6)	75.8 (6.8)	< 0.001
Ethnicity	Māori	412 (9.0%)	303 (5.7%)	209 (4.0%)	131 (3.2%)	35 (1.4%)	1090 (5.0%)	< 0.001
	Pacific peoples	120 (2.6%)	77 (1.5%)	74 (1.4%)	19 (0.5%)	8 (0.3%)	298 (1.4%)	
	Asian	178 (3.9%)	188 (3.5%)	148 (2.8%)	78 (1.9%)	46 (1.8%)	638 (2.9%)	
	European/Other	3879 (84.5%)	4741 (89.3%)	4797 (91.8%)	3834 (94.4%)	2445 (96.5%)	19,696 (90.7%)	
NZDep13 quintile	1 (lowest deprivation)	766 (16.7%)	809 (15.2%)	770 (14.7%)	552 (13.6%)	382 (15.1%)	3279 (15.1%)	0.003
	2	792 (17.3%)	896 (16.9%)	915 (17.5%)	724 (17.8%)	445 (17.6%)	3772 (17.4%)	
	3	971 (21.2%)	1202 (22.6%)	1156 (22.1%)	924 (22.7%)	570 (22.5%)	4823 (22.2%)	
	4	1079 (23.5%)	1288 (24.3%)	1351 (25.8%)	1044 (25.7%)	664 (26.2%)	5426 (25.0%)	
	5 (highest deprivation)	982 (21.4%)	1113 (21.0%)	1037 (19.8%)	819 (20.2%)	473 (18.7%)	4424 (20.4%)	
Cancer type	Colorectal	3841 (83.7%)	4622 (87.0%)	4716 (90.2%)	3833 (94.3%)	2465 (97.2%)	19,477 (89.6%)	< 0.001
	Stomach	258 (5.6%)	243 (4.6%)	215 (4.1%)	136 (3.3%)	31 (1.2%)	883 (4.1%)	
	Small intestine	125 (2.7%)	100 (1.9%)	99 (1.9%)	44 (1.1%)	32 (1.3%)	400 (1.8%)	
	Pancreas	117 (2.5%)	123 (2.3%)	64 (1.2%)	21 (0.5%)	2 (0.1%)	327 (1.5%)	
	Hepatic	91 (2.0%)	61 (1.1%)	36 (0.7%)	9 (0.2%)	1 (0.0%)	198 (0.9%)	
	Oesophagus	97 (2.1%)	89 (1.7%)	42 (0.8%)	9 (0.2%)	1 (0.0%)	238 (1.1%)	
	Biliary	61 (1.3%)	73 (1.4%)	58 (1.1%)	11 (0.3%)	3 (0.1%)	206 (0.9%)	
Acuity	Acute	946 (20.6%)	998 (18.8%)	1070 (20.5%)	898 (22.1%)	726 (28.6%)	4638 (21.3%)	< 0.001
	Elective	3644 (79.4%)	4313 (81.2%)	4160 (79.5%)	3165 (77.9%)	1809 (71.4%)	17,091 (78.7%)	
Cancer stage	Localized	1037 (27.2%)	1394 (30.4%)	1442 (31.0%)	1164 (31.1%)	660 (27.9%)	5697 (29.8%)	< 0.001
	Local invasion	765 (20.1%)	1017 (22.2%)	1121 (24.1%)	968 (25.8%)	679 (28.7%)	4550 (23.8%)	
	Regional nodes	1465 (38.5%)	1603 (35.0%)	1581 (34.0%)	1275 (34.0%)	804 (34.0%)	6728 (35.1%)	
	Distant	539 (14.2%)	569 (12.4%)	506 (10.9%)	338 (9.0%)	222 (9.4%)	2174 (11.4%)	
C3 comorbidity score	Mean (SD)	0.9 (1.5)	1.0 (1.6)	1.3 (1.8)	1.5 (1.9)	1.5 (1.9)	1.2 (1.7)	< 0.001
	0	1616 (35.2%)	1747 (32.9%)	1542 (29.5%)	992 (24.4%)	610 (24.1%)	6507 (29.9%)	< 0.001
	0–1	1776 (38.7%)	2015 (37.9%)	1786 (34.1%)	1320 (32.5%)	800 (31.6%)	7697 (35.4%)	
	1–2	500 (10.9%)	633 (11.9%)	698 (13.3%)	614 (15.1%)	388 (15.3%)	2833 (13.0%)	
	2–3	293 (6.4%)	352 (6.6%)	469 (9.0%)	421 (10.4%)	281 (11.1%)	1816 (8.4%)	
	> 3	405 (8.8%)	564 (10.6%)	735 (14.1%)	716 (17.6%)	456 (18.0%)	2876 (13.2%)	
Hospital frailty risk score	Mean (SD)	1.8 (3.7)	2.1 (4.3)	2.8 (5.1)	3.7 (5.9)	4.7 (6.8)	2.8 (5.1)	< 0.001
	Low risk (< 5)	4069 (88.6%)	4570 (86.0%)	4219 (80.7%)	3023 (74.4%)	1749 (69.0%)	17,630 (81.1%)	< 0.001
	Intermediate risk (5–15)	445 (9.7%)	621 (11.7%)	823 (15.7%)	818 (20.1%)	568 (22.4%)	3275 (15.1%)	
	High risk (> 15%)	76 (1.7%)	120 (2.3%)	188 (3.6%)	222 (5.5%)	218 (8.6%)	824 (3.8%)	

### 3.2 Postoperative Outcomes

Table 2 summarizes the unadjusted clinical outcomes. The overall 90‐day mortality rate was 6.1% (*n* = 1316), rising from 3.4% among patients aged 65–69 years to 11.9% among those ≥ 85 years (*p* < 0.001). Similarly, one‐year and two‐year mortality rates increased from 10.6% to 19.6% in the youngest group to 24.1% and 35.6% in the oldest group (both *p* < 0.001).

**TABLE 2:** Postoperative outcomes for the included cohort, stratified by chronological age.

	Age (years)						
Outcome	65–69	70–74	75–79	80–84	≥ 85	Total	*p*
Total *N* (%)	4590 (21.1%)	5311 (24.4%)	5230 (24.1%)	4063 (18.7%)	2535 (11.7%)	21,729	
Overall complications	2007 (43.7%)	2511 (47.3%)	2634 (50.4%)	2143 (52.7%)	1425 (56.2%)	10,720 (49.3%)	< 0.001
Reoperation	499 (10.9%)	557 (10.5%)	602 (11.5%)	400 (9.8%)	201 (7.9%)	2259 (10.4%)	< 0.001
Nonoperative complications	1508 (32.9%)	1954 (36.8%)	2032 (38.9%)	1743 (42.9%)	1224 (48.3%)	8461 (38.9%)	< 0.001
Percutaneous intervention	244 (5.3%)	265 (5.0%)	228 (4.4%)	149 (3.7%)	75 (3.0%)	961 (4.4%)	< 0.001
Endoscopic intervention	167 (3.6%)	166 (3.1%)	204 (3.9%)	118 (2.9%)	53 (2.1%)	708 (3.3%)	< 0.001
Blood transfusion	493 (10.7%)	656 (12.4%)	661 (12.6%)	570 (14.0%)	345 (13.6%)	2725 (12.5%)	< 0.001
Acute kidney injury	338 (7.4%)	488 (9.2%)	528 (10.1%)	437 (10.8%)	287 (11.3%)	2078 (9.6%)	< 0.001
Cardiac arrest	35 (0.8%)	29 (0.5%)	50 (1.0%)	35 (0.9%)	16 (0.6%)	165 (0.8%)	0.14
Cardiac arrythmia	306 (6.7%)	459 (8.6%)	502 (9.6%)	481 (11.8%)	324 (12.8%)	2072 (9.5%)	< 0.001
Myocardial infarction	70 (1.5%)	123 (2.3%)	160 (3.1%)	180 (4.4%)	159 (6.3%)	692 (3.2%)	< 0.001
Sepsis	242 (5.3%)	309 (5.8%)	290 (5.5%)	207 (5.1%)	130 (5.1%)	1178 (5.4%)	0.52
Shock	23 (0.5%)	35 (0.7%)	37 (0.7%)	21 (0.5%)	12 (0.5%)	128 (0.6%)	0.52
VTE	106 (2.3%)	149 (2.8%)	142 (2.7%)	100 (2.5%)	53 (2.1%)	550 (2.5%)	0.26
Pneumonia	331 (7.2%)	461 (8.7%)	535 (10.2%)	477 (11.7%)	350 (13.8%)	2154 (9.9%)	< 0.001
Respiratory failure	132 (2.9%)	178 (3.4%)	180 (3.4%)	145 (3.6%)	89 (3.5%)	724 (3.3%)	0.39
Stroke	23 (0.5%)	43 (0.8%)	42 (0.8%)	51 (1.3%)	44 (1.7%)	203 (0.9%)	< 0.001
Delirium	240 (5.2%)	403 (7.6%)	555 (10.6%)	524 (12.9%)	351 (13.8%)	2073 (9.5%)	< 0.001
Gastrointestinal bleeding	126 (2.7%)	148 (2.8%)	216 (4.1%)	165 (4.1%)	117 (4.6%)	772 (3.6%)	< 0.001
Surgical site infection	720 (15.7%)	831 (15.6%)	776 (14.8%)	546 (13.4%)	315 (12.4%)	3188 (14.7%)	< 0.001
Hemorrhage	208 (4.5%)	261 (4.9%)	266 (5.1%)	204 (5.0%)	120 (4.7%)	1059 (4.9%)	0.74
In‐hospital mortality	77 (1.7%)	122 (2.3%)	166 (3.2%)	210 (5.2%)	196 (7.7%)	771 (3.5%)	< 0.001
30‐day mortality	83 (1.8%)	132 (2.5%)	191 (3.7%)	231 (5.7%)	222 (8.8%)	859 (4.0%)	< 0.001
90‐day mortality	158 (3.4%)	234 (4.4%)	291 (5.6%)	331 (8.1%)	302 (11.9%)	1316 (6.1%)	< 0.001
1‐year mortality	487 (10.6%)	654 (12.3%)	761 (14.6%)	740 (18.2%)	611 (24.1%)	3253 (15.0%)	< 0.001
2‐year mortality	900 (19.6%)	1118 (21.1%)	1264 (24.2%)	1183 (29.1%)	903 (35.6%)	5368 (24.7%)	< 0.001
ICU admission	702 (15.3%)	827 (15.6%)	835 (16.0%)	666 (16.4%)	392 (15.5%)	3422 (15.7%)	0.65
Postoperative length of stay, median (IQR)	9.0 (6.0–13.0)	9.0 (6.0–13.0)	9.0 (7.0–14.0)	9.0 (7.0–14.0)	10.0 (7.0–14.0)	9.0 (7.0–14.0)	< 0.001
90‐Day unplanned readmission	1254 (27.3%)	1408 (26.5%)	1292 (24.7%)	904 (22.2%)	523 (20.6%)	5381 (24.8%)	< 0.001

Overall, 49.3% of patients experienced at least one postoperative complication within 90 days. This proportion increased steadily with age, from 43.7% in those 65–69 years to 56.2% in those ≥ 85 years (*p* < 0.001). Older age was associated with fewer reoperations (7.9% ≥ 85 years vs. 10.9% in 65–69 years), yet higher mortality once reoperation became necessary (22.9% FTR among reoperated patients ≥ 85 years vs. 9.6% among reoperated patients 65–69 years).

Failure to rescue (FTR), defined as death after any postoperative complication, occurred in 12.3% of patients experiencing complications (*n* = 1316/10,720). Older age groups had disproportionately higher FTR, from 7.9% in the youngest group to 21.2% in the oldest (*p* < 0.001). This age‐related gradient in FTR rates persisted for both nonoperative and surgical complications (Table 2) and was consistent across almost all analyzed individual complications (Table 3). Of note, although overall rates of complications were also higher in those with significant comorbidity or frailty, the greatest univariate difference in mortality and FTR was observed across age strata (Figure 1).

**TABLE 3:** Failure to rescue rates for each postoperative complication, stratified by age groups.

Complication	65–69	70–74	75–79	80–84	≥ 85	Total	*p* ^a^
Overall	158/2007 (7.87%)	234/2511 (9.32%)	291/2634 (11.05%)	331/2143 (15.45%)	302/1425 (21.19%)	1316/10,720 (12.28%)	< 0.001
Reoperation	48/499 (9.62%)	62/557 (11.13%)	79/602 (13.12%)	68/400 (17%)	46/201 (22.89%)	303/2259 (13.41%)	< 0.001
Nonoperative complication	110/1508 (7.29%)	172/1954 (8.8%)	212/2032 (10.43%)	263/1743 (15.09%)	256/1224 (20.92%)	1013/8461 (11.97%)	< 0.001
Percutaneous reintervention	22/244 (9.02%)	21/265 (7.92%)	28/228 (12.28%)	23/149 (15.44%)	13/75 (17.33%)	107/961 (11.13%)	0.004
Endoscopic reintervention	12/167 (7.19%)	18/166 (10.84%)	21/204 (10.29%)	13/118 (11.02%)	10/53 (18.87%)	74/708 (10.45%)	0.05
Blood transfusion	46/493 (9.33%)	75/656 (11.43%)	72/661 (10.89%)	81/570 (14.21%)	58/345 (16.81%)	332/2725 (12.18%)	< 0.001
Acute kidney injury	44/338 (13.02%)	67/488 (13.73%)	84/528 (15.91%)	102/437 (23.34%)	95/287 (33.1%)	392/2078 (18.86%)	< 0.001
Cardiac arrest	21/35 (60%)	20/29 (68.97%)	35/50 (70%)	26/35 (74.29%)	12/16 (75%)	114/165 (69.09%)	0.18
Cardiac arrhythmia	39/306 (12.75%)	52/459 (11.33%)	68/502 (13.55%)	84/481 (17.46%)	75/324 (23.15%)	318/2072 (15.35%)	< 0.001
Myocardial infarction	12/70 (17.14%)	30/123 (24.39%)	36/160 (22.5%)	49/180 (27.22%)	50/159 (31.45%)	177/692 (25.58%)	0.02
Sepsis	46/242 (19.01%)	63/309 (20.39%)	71/290 (24.48%)	79/207 (38.16%)	60/130 (46.15%)	319/1178 (27.08%)	< 0.001
Shock	9/23 (39.13%)	14/35 (40%)	14/37 (37.84%)	11/21 (52.38%)	5/12 (41.67%)	53/128 (41.41%)	0.56
Venous thromboembolism	14/106 (13.21%)	19/149 (12.75%)	23/142 (16.2%)	20/100 (20%)	8/53 (15.09%)	84/550 (15.27%)	0.22
Pneumonia	42/331 (12.69%)	59/461 (12.8%)	83/535 (15.51%)	110/477 (23.06%)	111/350 (31.71%)	405/2154 (18.8%)	< 0.001
Respiratory failure	40/132 (30.3%)	53/178 (29.78%)	54/180 (30%)	74/145 (51.03%)	47/89 (52.81%)	268/724 (37.02%)	< 0.001
Stroke	4/23 (17.39%)	11/43 (25.58%)	8/42 (19.05%)	18/51 (35.29%)	15/44 (34.09%)	56/203 (27.59%)	0.07
Delirium	20/240 (8.33%)	38/403 (9.43%)	50/555 (9.01%)	89/524 (16.98%)	58/351 (16.52%)	255/2073 (12.3%)	< 0.001
Gastrointestinal bleeding	13/126 (10.32%)	23/148 (15.54%)	27/216 (12.5%)	24/165 (14.55%)	15/117 (12.82%)	102/772 (13.21%)	0.67
Surgical site infection	37/720 (5.14%)	50/831 (6.02%)	61/776 (7.86%)	52/546 (9.52%)	42/315 (13.33%)	242/3188 (7.59%)	< 0.001
Hemorrhage	16/208 (7.69%)	20/261 (7.66%)	26/266 (9.77%)	20/204 (9.8%)	12/120 (10%)	94/1059 (8.88%)	0.28
ICU admission	56/702 (7.98%)	79/827 (9.55%)	111/835 (13.29%)	107/666 (16.07%)	84/392 (21.43%)	437/3422 (12.77%)	< 0.001

^a^ *p* value represents univariate ordinal chi‐squared test between age groups.

On univariate analysis, increasing age, comorbidity, and frailty were all strongly correlated with higher rates of mortality, complications, and FTR (all *p* < 0.001 and Figure 1). Older patients had a significantly higher unadjusted rates of postoperative mortality (per 5‐year increase, univariate OR 1.40, 95% CI 1.34–1.45, and *p* < 0.001). This was primarily driven by a higher rate of FTR (univariate OR 1.33, 95% CI 1.28–1.39, and *p* < 0.001) compared with a smaller difference in postoperative complications (univariate OR 1.13, 95% CI 1.11–1.15, and *p* < 0.001).

### 3.3 Multivariable Analysis

**FIGURE 2:**
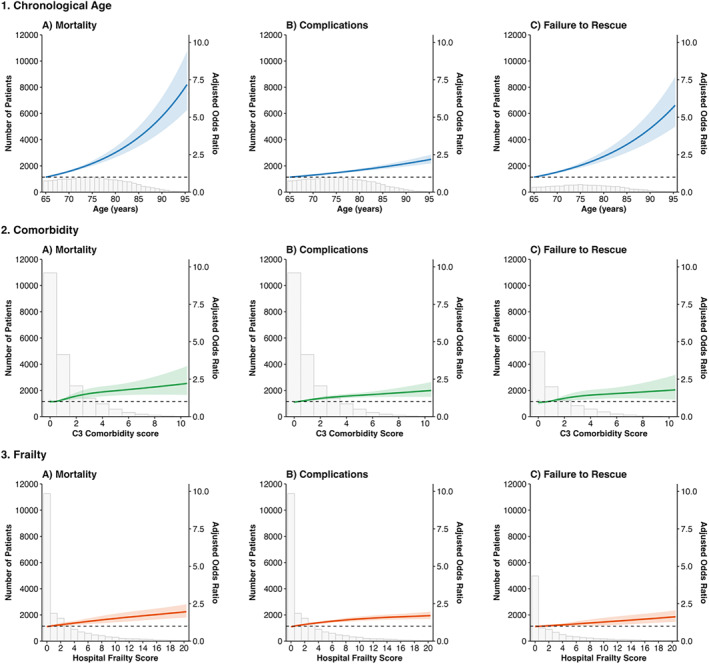
Impact of chronological age, comorbidity, and frailty on mortality, complications, and failure to rescue. Model lines and shaded area represent adjusted odds ratio and 95% confidence interval. An exponential model was fitted for age and restricted cubic splines models for comorbidity and frailty. The horizontal dashed line represents the reference value. The histogram shows the distribution of patients in the cohort according to the explanatory variable.

Table 4 and Figure 2 depict the adjusted associations of age, comorbidity, and frailty with postoperative mortality, complications, and FTR. When modeled as continuous variables, each additional five years of age was associated with a 40% increase in odds of 90‐day mortality (aOR 1.40, 95% CI 1.34–1.46, and *p* < 0.001) and a 35% increase in odds of FTR (aOR 1.35, 95% CI 1.29–1.41, and *p* < 0.001). By contrast, elevated comorbidity and frailty conferred more modest but still significant risks for mortality and complications. For example, compared with low frailty risk, patients at high frailty risk had more than double the adjusted odds of 90‐day mortality (aOR 2.04, 95% CI 1.52–2.74, and *p* < 0.001) and 73% increased odds of FTR (aOR 1.73, 95% CI 1.27–2.35, and *p* = 0.001). Nonetheless, chronological age emerged as the most pronounced independent driver of postoperative death and FTR, even after accounting for comorbidity and frailty (Figure 2). Full models and odds ratios are shown in Supporting Information S1: Table 3.

**TABLE 4:** Multivariate logistic regression results demonstrating the impact of age, comorbidty, and frailty on 90‐day postoperative mortality, complications, and failure to rescue.

Variable	Mortality		Complications		FTR	
	aOR (95% CI)	*p*	aOR (95% CI)	*p*	aOR (95% CI)	*p*
Age (per year)	1.07 (1.06–1.08)	< 0.001	1.03 (1.02–1.03)	< 0.001	1.06 (1.05–1.07)	< 0.001
Age (per 5 years)	1.40 (1.34–1.46)	< 0.001	1.15 (1.12–1.17)	< 0.001	1.35 (1.29–1.41)	< 0.001
Age (per 10 years)	1.96 (1.80–2.14)	< 0.001	1.31 (1.26–1.37)	< 0.001	1.81 (1.65–1.99)	< 0.001
Comorbidity (0 vs. 0–1)	0.99 (0.84–1.17)	0.92	0.89 (0.83–0.96)	0.002	1.05 (0.88–1.25)	0.61
Comorbidity (0 vs. 1–2)	1.40 (1.15–1.70)	0.001	1.16 (1.06–1.27)	0.001	1.33 (1.09–1.62)	0.01
Comorbidity (0 vs. 2–3)	1.55 (1.25–1.93)	< 0.001	1.36 (1.22–1.51)	< 0.001	1.42 (1.14–1.77)	0.002
Comorbidity (0 vs. > 3)	1.98 (1.64–2.38)	< 0.001	1.53 (1.39–1.68)	< 0.001	1.75 (1.44–2.11)	< 0.001
Frailty (low vs. intermediate)	1.33 (1.12–1.58)	0.001	1.41 (1.28–1.56)	< 0.001	1.17 (0.98–1.40)	0.08
Frailty (low vs. high)	2.04 (1.52–2.74)	< 0.001	1.70 (1.36–2.12)	< 0.001	1.73 (1.27–2.35)	0.001

*Note:* Other variables included in logistic regression models: sex, ethnicity, socioeconomic deprivation, admission acuity, cancer type, cancer extent, and operation year.

Abbreviations: 95% CI, 95% confidence interval; aOR, adjusted odds ratio.

### 3.4 Subgroup Analyses

Separate analysis stratified for patients with colorectal cancer and with all other cancer types demonstrated no meaningful differences to the overall analysis (Supporting Information S1: Figures 2–4).

## 4 Discussion

In this nationwide cohort of older adults undergoing gastrointestinal and hepatobiliary cancer surgery, we found that increasing chronological age, comorbidity burden, and frailty risk were each independently associated with significantly worse postoperative outcomes. Notably, chronological age emerged as the strongest predictor of both 90‐day mortality and FTR, even after adjustment for comorbidity, frailty, and other confounders. Although the incidence of complications increased modestly with age, the most striking age‐related gradient was observed in FTR rates, underscoring a critical vulnerability of older adults to postoperative deterioration and death once complications occur. Traditionally, hospital‐level factors have been emphasized as drivers of center‐level variation in FTR [22, 23]. However, this study also highlights the impact of patient‐level factors on FTR and other surgical outcomes, and the need for careful risk adjustment when reporting hospital outcomes.

These findings align with and extend previous work by Beier et al. who identified older age was a stronger predictor of 30‐day FTR following GI cancer surgery than either comorbidity or frailty as defined by the Risk Adjustment Index [24]. Although comorbidity and frailty are well‐established predictors of poor surgical outcomes, our results suggest that neither fully captures the systemic physiological decline associated with aging that impairs recovery after major complications. This may reflect the multifactorial nature of aging, encompassing diminished cardiorespiratory reserve, immune dysfunction, impaired inflammatory response, and cognitive or social vulnerability, all of which contribute to reduced capacity to survive adverse postoperative events [25]. Prior work has demonstrated that older patients are more likely to die following colorectal anastomotic leak [26], complications of major trauma [27], and postoperative pulmonary complications [28]. However, our work showed higher rates of FTR following almost all studied complications amongst older adults, suggesting this is a generalizable phenomenon across all postoperative adverse events.

These findings have important implications for perioperative risk stratification and optimization in older surgical patients. Comprehensive geriatric assessment models and structured programs, such as geriatric comanagement, prehabilitation, and initiatives, such as ACS NSQIP/AGS Strong for Surgery, aim to improve patient selection, optimize modifiable risk factors, and support shared decision‐making in this population [29]. However, our results also highlight potential limitations of administrative‐based frailty measures, such as the Hospital Frailty Risk Score, which may incompletely capture physiological vulnerability, and underscore the need for direct comparison with prospectively assessed clinical tools such as the Clinical Frailty Scale and other bedside measures of functional reserve [30]. This may partly explain why age emerged as a stronger predictor than frailty in this analysis, despite frailty demonstrating greater predictive value in some prospective studies [31]. However, even after accounting for comorbidity and frailty, chronological age remained a strong independent predictor of postoperative mortality and FTR in patients undergoing gastrointestinal cancer surgery. This suggests that even physiologically fit older patients should be considered at intrinsically higher perioperative risk, reinforcing the need for shared decision making, heightened surveillance, careful selection, and tailored perioperative strategies in this group [24].

These findings also highlight the importance of strategies to proactively prevent, recognize, and escalate care for postoperative complications, particularly amongst older surgical patients. Older adults are less likely to exhibit early physiological warning signs of deterioration [32] and may be more prone to atypical presentations of postoperative complications. Cognitive impairment, delirium, and medication‐related issues may further obscure clinical deterioration, contributing to delays in recognition and treatment [33]. Enhanced recovery programs (ERPs) [34, 35], routine preoperative frailty screening [36], and geriatric comanagement models have each shown promise in improving surgical outcomes and may also reduce FTR. Older patients also experience unique challenges in perioperative care, including nuanced medication management, increased risk of polypharmacy, and difficulties in mobilization and rehabilitation, which may be better addressed with shared geriatric input [37, 38]. Moreover, tailored prehabilitation, optimization of chronic conditions, and structured shared decision‐making are particularly important in this group. These strategies may not only reduce complication incidence but also improve patients' physiological resilience to survive them when they occur [39].

We observed a higher concentration of comorbidity among Māori patients, who were also underrepresented in the oldest age groups. This reflects the cumulative impact of systemic health inequities, resulting in a greater burden of chronic disease. Māori ethnicity has previously been identified as an independent risk factor for FTR [18], highlighting the need for targeted perioperative strategies that address both biological, social, and structural risk factors driving inequities in surgical outcomes.

There are several limitations to this analysis of linked administrative data, which has inherent potential biases due to misclassification or inaccuracies in identifying certain complications or comorbidities. We were limited by the lack of data on surgical technique, institutional care protocols, or surgeon volume, all of which may influence postoperative outcomes and may contribute to residual unmeasured confounding. We included the first eligible oncological operation per patient, to ensure statistical independence and avoid intraindividual correlation. However, this may have introduced bias by excluding patients with repeat operations, who may represent a high‐risk subgroup. Finally, our data were derived from a single national healthcare system, which may limit generalizability to other countries with different health infrastructures or patient demographics. We were unable to accurately assess admission to aged care facilities or changes in functional status as these data were not available; this remains an area requiring further research.

## 5 Conclusion

Chronological age, comorbidity, and frailty are each independently associated with postoperative mortality and FTR following gastrointestinal cancer surgery in older adults. Chronological age was the most dominant predictor of postoperative death, particularly among patients who experienced complications. These findings highlight the critical importance of recognizing older adults as a high‐risk group not only for complications but also for failure to recover once complications occur. Future research should evaluate whether structured perioperative interventions can reduce FTR rates and improve recovery for older surgical patients.

## Author Contributions

**Cameron I. Wells:** conceptualization, methodology, software, formal analysis, writing – original draft, funding acquisition. **Chris Varghese:** methodology, data curation, writing – review and editing. **Greg O'Grady:** supervision, writing – review and editing. **Ian P. Bissett:** supervision, writing – review and editing.

## Funding

C.W. is supported by a Health Research Council of New Zealand Clinical Research Training Fellowship (22/45).

## Conflicts of Interest

The authors declare no conflicts of interest.

## Supporting information


Supporting Information S1

## Data Availability

Data may be obtained from the New Zealand Ministry of Health National Collections team. Requests can be made by contacting data-enquiries@health.govt.nz. The code used for the analyses in this manuscript is available from the authors upon reasonable request.
