# Supplementary material for: Age, Frailty, and Comorbidity as Predictors of Mortality and Failure to Rescue After Gastrointestinal Cancer Surgery: A National Retrospective Cohort Study
Source: World J Surg. 2026 Feb 14;50(3):713–23. doi: 10.1002/wjs.70268 (PMC13006779; doi:10.1002/wjs.70268)

**Supplementary Table 1**. C3 Comorbidity Index Codes

| C3 Comorbidity Index |  |
| --- | --- |
| AIDS | B20, B21, B22, B23, B24, F02.4, Z21 |
| Alcohol abuse | F10.1, F10.2, F10.3, F10.4, F10.5, F10.6, F10.7, F10.8, F10.9, Z50.2, Z71.4 |
| Anemia | D50, D51, D52, D53 |
| Angina | I20 |
| Anxiety and behavioral disorders | F40, F41, F42, F44, F45, F48, F50, F55, F59, F60, F61, F63, F64, F65, F66, F68, F69 |
| Aortic and other aneurysms | I71, I72 |
| Bone disorders | M80, M83.0, M83.1, M83.2, M83.3, M83.4, M83.5, M83.8, M83.9, M85, M86.3, M86.4, M86.5, M86.6, M88 |
| Bowel disease: inflammatory | K50, K51, K52.2, K52.8, K52.9 |
| Breast cancer | C50 |
| Cardiac arrhythmia | I44.1, I44.2, I44.3, I45.6, I45.9, I47, I48, I49, T82.1, Z45.0, Z95.0 |
| Cardiac disease: other | I11.9, I24.8, I24.9, I25.0, I25.1, I25.3, I25.4, I25.6, I25.8, I25.9, I31.0, I31.1, I42.1, I42.2, I42.4 |
| Cardiac valve disease | I05, I06, I07, I08, I09.1, I09.8, I34, I35, I36, I37, I38, T82.0, Q23.0, Q23.1, Q23.2, Q23.3, Q23.8, Q23.9, Z95.2, Z95.3, Z95.4 |
| Cerebrovascular disease | I60, I61, I62, I63, I64, I65, I66, I67, I69, G45, G46 |
| Chronic pulmonary | E84, J40, J41, J42, J43, J44, J45, J46, J47, J60, J61, J62, J63, J64, J65, J66, J67, J68.4, J70.1, J70.3, J84, J96.1, J98.0, J98.2, J98.3, J98.4 |
| Chronic renal disease | I12.0, I12.9, I13.9, I13.1, N03.2, N03.3, N03.4, N03.5, N03.6, N03.7, N03.8, N03.9, N04.2, N04.3, N04.4, N04.5, N04.6, N04.7, N04.8, N04.9, N05.2, N05.3, N05.4, N05.5, N05.6, N05.7, N05.8, N05.9, N11, N18, N19, N25.0, N25.8, N25.9, Q60, Q61.1, Q61.2, Z49, Z94.0, Z99.2 |
| Coagulopathy and other blood disorders | D55, D56, D57, D58, D59.0, D59.1, D59.2, D59.3, D59.4, D59.8, D59.9, D60, D61, D64, D66, D67, D68.0, D68.1, D68.2, D68.8, D68.9, D69.1 , D69.2, D69.3, D69.4, D69.6, D69.8, D69.9, D70, D71, D72, D74, D75.0, D75.2, D75.8, D75.9 |
| Colorectal cancer | C18, C19, C20, C21 |
| Complications of diabetes | I20, I21, I22, I23, I24, I25,I60, I61, I62, I63, I64, I65, I66, I67, I68, I69, I70, I71, I72, I73, I74, I75, I76, I77, I78, I79, N03, N04, N18, G60.3, G62, G63.8, H35, H36, L97 |
| Congestive heart failure | I09.9, I11.0, I13.0, I13.2, I25.5, I42.0, I42.5, I42.6, I42.7, I42.8, I42.9, I43, I50 |
| Connective tissue disease | L93, M05, M06, M08, M12.0, M12.3, M30, M31, M32, M33, M34, M35.0, M35.1,M35.2, M35.3, M35.4, M35.5, M35.6, M35.8, M35.9 |
| Dementia | F00, F01, F02.0, F02.1, F02.2, F02.3, F03, F05.1, G30, G31.0, G31.1 |
| Diabetes (complicated) | E10.2, E10.3, E10.4, E10.5, E10.6, E10.7, E10.8, E11.2, E11.3, E11.4, E11.5, E11.6, E11.7, E11.8, E12.2, E12.3, E12.4, E12.5, E12.6, E12.7, E12.8, E13.2, E13.3, E13.4, E13.5, E13.6, E13.7, E13.8, E14.2, E14.3, E14.4, E14.5, E14.6, E14.7, E14.8 |
| Diabetes (uncomplicated) | E10.0, E10.1, E10.9, E11.0, E11.1, E11.9, E12.0, E12.1, E12.9, E13.0, E13.1, E13.9, E14.0, E14.1, E14.9 |
| Drug abuse | F11, F12, F13, F14, F15, F16, F18, F19, Z50.3, Z71.5, Z72.2 |
| Endocrine disorder | E01, E02, E03, E05, E06.2, E06.3, E06.5, E07, E16.3, E16.4, E16.8, E16.9, E20, E21.0, E21.2, E21.3, E21.4, E21.5, E22, E23.0, E23.2, E23.3, E23.6, E23.7, E24.0, E24.1, E24.3, E24.4, E24.8, E24.9, E25, E26, E27, E31, E32, E34.5, E34.8, E34.9 |
| Epilepsy | G40.0, G40.1, G40.2, G40.3, G40.4, G40.6, G40.7, G40.8, G40.9, G41 |
| Eye problem long term | H16, H18.1, H18.4, H18.5, H18.6, H20.1, H21.2, H30.1, H31.1, H31.2, H31.3, H31.4, H33.0, H33.2, H33.3, H33.4, H33.5, H34, H35, H43, H46, H47, H49, H50, H51, H53.0, H53.1, H53.2, H53.3, H53.4, H53.6,H53.8, H53.9, H54, Q12, Q13, Q14, Q15 |
| Gastrointestinal ulcer or upper GI disease | K22.0, K22.1, K22.4, K22.5, K22.8, K22.9, K25, K26, K27, K28, K31.1, K31.2, K31.4, K31.6 |
| Gynecological cancers | C51, C52, C53, C54, C55, C56, C57, C58 |
| Hepatitis, chronic viral | B18, B94.2, Z22.5 |
| Hypertension (uncomplicated) | I10 |
| Immune system disorder | D80, D81, D82, D83, D84, D86, D89 |
| Infection: Chronic NOS | A30, A31, A52, B91, B92, B94.1, B94.8, B94.9 |
| Inner ear disorder | H80, H81, H83, H90, H91.0, H91.1, H91.3, H91.8, H91.9, H93.0, H93.1, H93.2, H93.3 |
| Intestinal disorder | K57, K59.2, K59.3, K90 |
| Joint or spinal disorder | M07, M13, M15.0, M15.1, M15.2, M15.4, M15.8, M15.9, M40.0, M40.2, M40.3, M40.4, M40.5, M41, M42, M43, M45, M46.0, M46.1, M46.2, M47, M48.0, M48.1, M48.2, M48.5, M48.8, M48.9, G95.0, G95.1 |
| Liver disease (moderate or severe) | K70, K71.1, K71.3, K71.4, K71.5, K71.7, K72.1, K72.9, K73, K74, K76.0, K76.2, K76.3, K76.4, K76.5, K76.6, K76.7, K76.8, K76.9, I85, I86.4, I98.2, Z94.4 |
| Lung cancer | C33, C34 |
| Lymphomas and leukemias | C81, C82, C83, C84, C85, C91, C92, C93, C94, C95, C96 |
| Major psychiatric disorder | F20, F22, F25, F28, F29, F30.2, F31, F32.1, F32.2, F32.3, F32.8, F32.9, F33, F39 |
| Malignant melanoma | C43 |
| Malnutrition and other nutritional disorders | E40, E41, E42, E43, E44, E45, E46, E50, E51, E52, E53, E54, E55, E56, E58, E59, E60, E61, E63, E64 |
| Mental and behavioral disorders due to brain damage | F04, F06, F07.0, F07.1 , F07.8, F07.9, F09, G93.1 |
| Mental retardation | F70, F71, F72, F73, F78, F79, F84.2, F84.3, F84.4, E00.0, E00.1, E00.2, E00.9, Q90 |
| Metabolic disorder | E70, E71, E72, E74, E75, E76, E77, E78, E79.1, E79.8, E79.9, E80, E83, E85, E88 |
| Metastatic cancer | C77,C78,C79 |
| Muscular peripheral nerve disorders | G60, G61, G62.0, G62.1, G62.2, G62.8, G62.9, G64, G70, G71, G72.0, G72.1, G72.2, G72.3, G72.4, G72.8, G72.9, G73.1 |
| Myocardial infarction | I21, I22, I23, I24.1, I25.2 |
| Obesity | E66 |
| Osteoporosis (uncomplicated) | M81.0, M81.1, M81.5, M81.8, M81.9 |
| Other cancers | C0, C10, C11, C12, C13, C14, C15, C16, C17, C18, C19,C20, C21, C23, C24, C25, C26, C30, C31, C32, C33, C37, C38, C39, C43, C45, C46, C47, C48, C49, C50, C51, C52, C53, C54, C55, C56, C57, C58,  C60, C61, C62, C63, C64, C65, C66, C67, C68, C69, C70, C72, C73, C74, C75, C81, C82, C83, C84, C85, C88, C90, C91, C92, C93, C94, C95 |
| Other neurologic disorders (excluding epilepsy) | G10, G11.0, G11.1, G11.2, G11.3, G11.8, G11.9, G12, G13, G20, G21, G23, G25.5, G31.2, G31.8, G31.9, G35, G36, G37, G90, G93.4, R47.0 |
| Pancreatitis | K85.0, K86.0, K86.1, K86.8 |
| Paralysis | G04.1, G11.4, G80.0, G80.1, G80.2, G81, G82, G83.0, G83.1, G83.2, G83.3, G83.4, G83.9 |
| Peripheral vascular disease | I70, I73.1, I73.8, I73.9, I74, I77.1, K55.1, K55.2, K55.8, K55.9 |
| Prostate cancer | C61 |
| Pulmonary circulation disorders | I26, I27, I28.0, I28.1, I28.8, I28.9 |
| Sleep disorder | F51, G47.0, G47.1, G47.2, G47.3 |
| Tuberculosis | A15, A16, A17, A18, A19, B90 |
| Upper gastrointestinal cancer | C15, C16, C17, C22, C23, C24, C25 |
| Urinary tract problem (chronic) | N30.1, N30.2, N31, N32, N35, N36 |
| Venous insufficiency | I83.0, I83.2, I87.2 |

**Supplementary Table 2**. Hospital Frailty Risk Score Codes

| Other bacterial intestinal infections | A04 |
| --- | --- |
| Diarrhoea and gastroenteritis of presumed infectious origin | A09 |
| Other septicaemia | A41 |
| Streptococcus and staphylococcus as the cause of diseases classified to other chapters | B95 |
| Other bacterial agents as the cause of diseases classified to other chapters (secondary code) | B96 |
| Other anaemias | D64 |
| Thyrotoxicosis [hyperthyroidism] | E05 |
| Other disorders of pancreatic internal secretion | E16 |
| Deficiency of other B group vitamins | E53 |
| Vitamin D deficiency | E55 |
| Disorders of mineral metabolism | E83 |
| Volume depletion | E86 |
| Other disorders of fluid, electrolyte and acid- base balance | E87 |
| Dementia in Alzheimer's disease | F00 |
| Vascular dementia | F01 |
| Unspecified dementia | F03 |
| Delirium, not induced by alcohol and other psychoactive substances | F05 |
| Mental and behavioural disorders due to use of alcohol | F10 |
| Depressive episode | F32 |
| Parkinson's disease | G20 |
| Alzheimer's disease | G30 |
| Other degenerative diseases of nervous system, not elsewhere classified | G31 |
| Epilepsy | G40 |
| Transient cerebral ischaemic attacks and related syndromes | G45 |
| Hemiplegia | G81 |
| Blindness and low vision | H54 |
| Other hearing loss | H91 |
| Cerebral Infarction | I63 |
| Other cerebrovascular diseases | I67 |
| Sequelae of cerebrovascular disease (secondary codes) | I69 |
| Hypotension | I95 |
| Pneumonia, organism unspecified | J18 |
| Unspecified acute lower respiratory infection | J22 |
| Pneumonitis due to solids and liquids | J69 |
| Respiratory failure, not elsewhere classified | J96 |
| Duodenal ulcer | K26 |
| Other noninfective gastroenteritis and colitis | K52 |
| Other functional intestinal disorders | K59 |
| Other diseases of digestive system | K92 |
| Cellulitis | L03 |
| Other local infections of skin and subcutaneous tissue | L08 |
| Decubitus ulcer | L89 |
| Ulcer of lower limb, not elsewhere classified | L97 |
| Polyarthrosis | M15 |
| Other arthrosis | M19 |
| Other joint disorders, not elsewhere classified | M25 |
| Scoliosis | M41 |
| Spinal stenosis (secondary code only) | M48 |
| Other soft tissue disorders, not elsewhere classified | M79 |
| Osteoporosis with pathological fracture | M80 |
| Osteoporosis without pathological fracture | M81 |
| Acute renal failure | N17 |
| Chronic renal failure | N18 |
| Unspecified renal failure | N19 |
| Calculus of kidney and ureter | N20 |
| Other disorders of kidney and ureter, not elsewhere classified | N28 |
| Other disorders of urinary system (includes urinary tract infection and urinary incontinence) | N39 |
| Abnormalities of heart beat | R00 |
| Gangrene, not elsewhere classified | R02 |
| Nausea and vomiting | R11 |
| Dysphagia | R13 |
| Abnormalities of gait and mobility | R26 |
| Other symptoms and signs involving the nervous and musculoskeletal systems (R29·6 Tendency to fall) | R29 |
| Unspecified haematuria | R31 |
| Unspecified urinary incontinence | R32 |
| Retention of urine | R33 |
| Somnolence, stupor and coma | R40 |
| Other symptoms and signs involving cognitive functions and awareness | R41 |
| Other symptoms and signs involving general sensations and perceptions | R44 |
| Symptoms and signs involving emotional state | R45 |
| Speech disturbances, not elsewhere classified | R47 |
| Fever of unknown origin | R50 |
| Senility | R54 |
| Syncope and collapse | R55 |
| Convulsions, not elsewhere classified | R56 |
| Symptoms and signs concerning food and fluid intake | R63 |
| Unknown and unspecified causes of morbidity | R69 |
| Other abnormal findings of blood chemistry | R79 |
| Abnormal results of function studies | R94 |
| Superficial injury of head | S00 |
| Open wound of head | S01 |
| Intracranial injury | S06 |
| Other and unspecified injuries of head | S09 |
| Fracture of rib(s), sternum and thoracic spine | S22 |
| Fracture of lumbar spine and pelvis | S32 |
| Fracture of shoulder and upper arm | S42 |
| Open wound of forearm | S51 |
| Fracture of femur | S72 |
| Superficial injury of lower leg | S80 |
| Complications of genitourinary prosthetic devices, implants and grafts | T83 |
| Agent resistant to penicillin and related antibiotics | U80 |
| Fall on same level from slipping, tripping and stumbling | W01 |
| Fall involving bed | W06 |
| Fall on and from stairs and steps | W10 |
| Other fall on same level | W18 |
| Unspecified fall | W19 |
| Exposure to unspecified factor | X59 |
| Other medical procedures as the cause of abnormal reaction of the patient | Y84 |
| Nosocomial condition | Y95 |
| Carrier of infectious disease | Z22 |
| Care involving use of rehabilitation procedures | Z50 |
| Problems related to social environment | Z60 |
| Problems related to life-management difficulty | Z73 |
| Problems related to care-provider dependency | Z74 |
| Problems related to medical facilities and other health care | Z75 |
| Personal history of other diseases and conditions | Z87 |
| Personal history of risk-factors, not elsewhere classified | Z91 |
| Artificial opening status | Z93 |
| Dependence on enabling machines and devices | Z99 |

Supplementary Table 3. Multivariable predictors of 90-day postoperative mortality, complications, and failure to rescue.

|  | **Mortality** | | **Complications** | | | | **FTR** | |
| --- | --- | --- | --- | --- | --- | --- | --- | --- |
| **Variable** | **OR (95% CI)** | **P** | | **OR (95% CI)** | **P** | **OR (95% CI)** | | **P** |
| **Age**  (per 5 years) | 1.40 (1.34-1.46) | <0.001 | | 1.10 (1.09-1.11) | <0.001 | 1.35 (1.29-1.41) | | <0.001 |
| **Female Sex**  (versus Male) | 0.82 (0.74-0.92) | <0.001 | | 0.72 (0.69-0.75) | <0.001 | 0.93 (0.83-1.05) | | 0.23 |
| **Māori**  (versus European/Other) | 1.58 (1.29-1.92) | <0.001 | | 1.34 (1.22-1.47) | <0.001 | 1.38 (1.13-1.70) | | 0.002 |
| **Pacific Peoples**  (versus European/Other) | 0.99 (0.66-1.46) | 0.94 | | 1.39 (1.20-1.62) | <0.001 | 0.82 (0.55-1.22) | | 0.33 |
| **Asian**  (versus European/Other) | 0.73 (0.50-1.06) | 0.1 | | 1.06 (0.94-1.19) | 0.36 | 0.70 (0.48-1.03) | | 0.07 |
| **NZDep Quintile 2**  (versus 1) | 0.98 (0.80-1.21) | 0.86 | | 1.05 (0.97-1.14) | 0.24 | 0.96 (0.78-1.19) | | 0.71 |
| **NZDep Quintile 3**  (versus 1) | 1.00 (0.82-1.21) | 0.99 | | 1.03 (0.95-1.11) | 0.5 | 0.97 (0.79-1.19) | | 0.78 |
| **NZDep Quintile 4**  (versus 1) | 1.18 (0.98-1.41) | 0.09 | | 1.05 (0.97-1.13) | 0.22 | 1.16 (0.96-1.40) | | 0.13 |
| **NZDep Quintile 5**  (versus 1) | 1.33 (1.10-1.61) | 0.003 | | 1.12 (1.03-1.21) | 0.01 | 1.32 (1.09-1.61) | | 0.005 |
| **Acute Surgery**  (versus Elective) | 3.05 (2.71-3.43) | <0.001 | | 1.81 (1.71-1.93) | <0.001 | 2.49 (2.21-2.82) | | <0.001 |
| **C3 0-1**  (versus 0) | 1.10 (0.94-1.28) | 0.23 | | 1.15 (1.09-1.22) | <0.001 | 1.04 (0.89-1.22) | | 0.65 |
| **C3 1-2**  (versus 0) | 1.46 (1.22-1.76) | <0.001 | | 1.33 (1.23-1.45) | <0.001 | 1.30 (1.08-1.58) | | 0.01 |
| **C3 2-3**  (versus 0) | 1.67 (1.35-2.06) | <0.001 | | 1.63 (1.47-1.80) | <0.001 | 1.43 (1.15-1.78) | | 0.001 |
| **C3 >3**  (versus 0) | 1.96 (1.62-2.38) | <0.001 | | 1.73 (1.58-1.91) | <0.001 | 1.63 (1.34-1.99) | | <0.001 |
| **Stomach Cancer**  (vs Colorectal) | 1.50 (1.17-1.93) | 0.002 | | 1.70 (1.53-1.89) | <0.001 | 1.25 (0.96-1.61) | | 0.09 |
| **Small Intestine Cancer**  (vs Colorectal) | 0.87 (0.62-1.22) | 0.42 | | 0.76 (0.65-0.88) | <0.001 | 1.02 (0.72-1.47) | | 0.9 |
| **Pancreas Cancer**  (vs Colorectal) | 2.35 (1.66-3.33) | <0.001 | | 2.29 (1.96-2.69) | <0.001 | 1.66 (1.16-2.37) | | 0.01 |
| **Hepatic Cancer**  (vs Colorectal) | 2.08 (1.33-3.25) | 0.001 | | 1.21 (1.02-1.45) | 0.03 | 2.11 (1.34-3.33) | | 0.001 |
| **Oesphagus Cancer**  (vs Colorectal) | 2.98 (1.98-4.47) | <0.001 | | 3.76 (3.06-4.61) | <0.001 | 1.85 (1.23-2.78) | | 0.003 |
| **Biliary Cancer**  (vs Colorectal) | 2.46 (1.58-3.82) | <0.001 | | 3.66 (2.91-4.62) | <0.001 | 1.58 (1.01-2.47) | | 0.045 |
| **Local Invasion**  (versus Localised) | 1.52 (1.27-1.82) | <0.001 | | 1.03 (0.96-1.10) | 0.43 | 1.52 (1.27-1.83) | | <0.001 |
| **Regional Nodes**  (versus Localised) | 1.66 (1.42-1.96) | <0.001 | | 1.13 (1.06-1.19) | <0.001 | 1.61 (1.36-1.90) | | <0.001 |
| **Distant Metastases**  (versus Localised) | 5.00 (4.20-5.96) | <0.001 | | 1.54 (1.42-1.67) | <0.001 | 4.60 (3.84-5.52) | | <0.001 |
| **2009-12**  (versus 2005-08) | 0.86 (0.75-0.99) | 0.04 | | 0.96 (0.90-1.03) | 0.24 | 0.89 (0.77-1.03) | | 0.11 |
| **2013-16**  (versus 2005-08) | 0.62 (0.54-0.72) | <0.001 | | 0.88 (0.83-0.94) | <0.001 | 0.65 (0.55-0.75) | | <0.001 |
| **2017-20**  (versus 2005-08) | 0.56 (0.47-0.65) | <0.001 | | 0.76 (0.71-0.81) | <0.001 | 0.62 (0.52-0.73) | | <0.001 |
| **Intermediate Frailty Risk** (versus Low Risk) | 1.32 (1.13-1.53) | <0.001 | | 1.35 (1.25-1.45) | <0.001 | 1.19 (1.01-1.39) | | 0.03 |
| **High Frailty Risk**  (versus Low Risk) | 2.04 (1.52-2.74) | <0.001 | | 1.69 (1.45-1.98) | <0.001 | 1.73 (1.27-2.35) | | <0.001 |

**Supplementary Figure 1.** Flow diagram of participant inclusion.
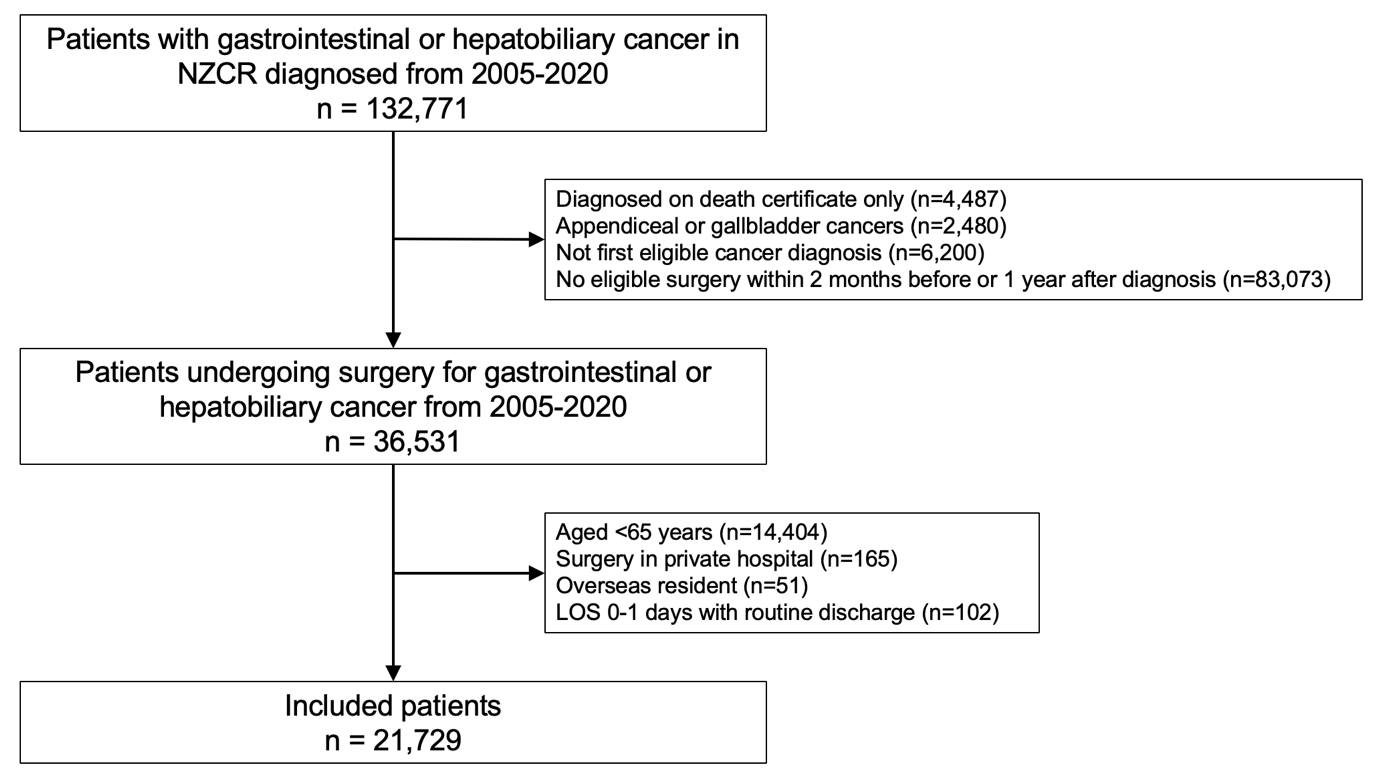


**Supplementary Figure 2.** Impact of chronological age, comorbidity, and frailty on mortality, complications, and failure to rescue amongst patients with colorectal cancer.


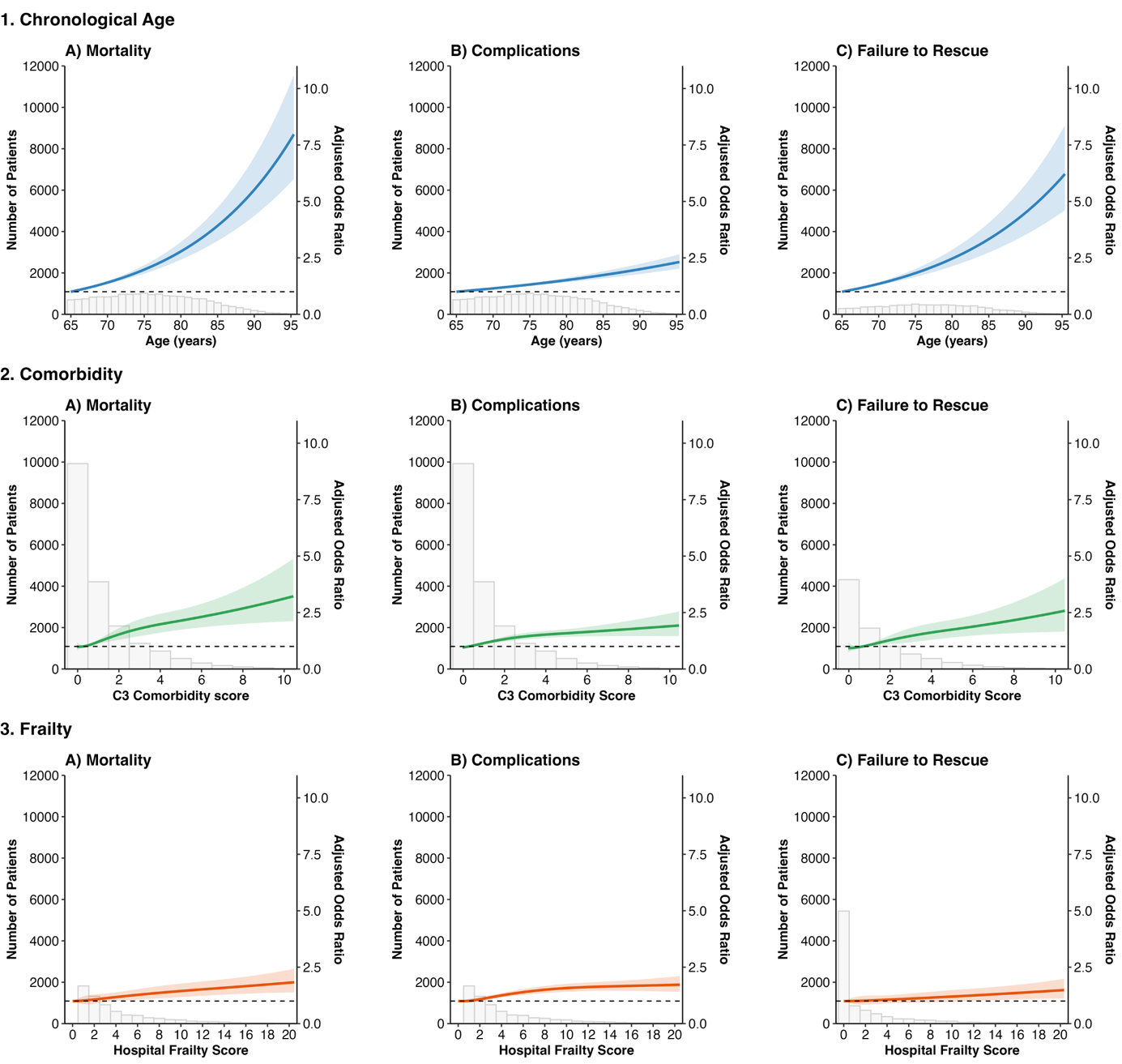


**Supplementary Figure 3.** Impact of chronological age, comorbidity, and frailty on mortality, complications, and failure to rescue amongst patients with cancer types other than colorectal cancer.


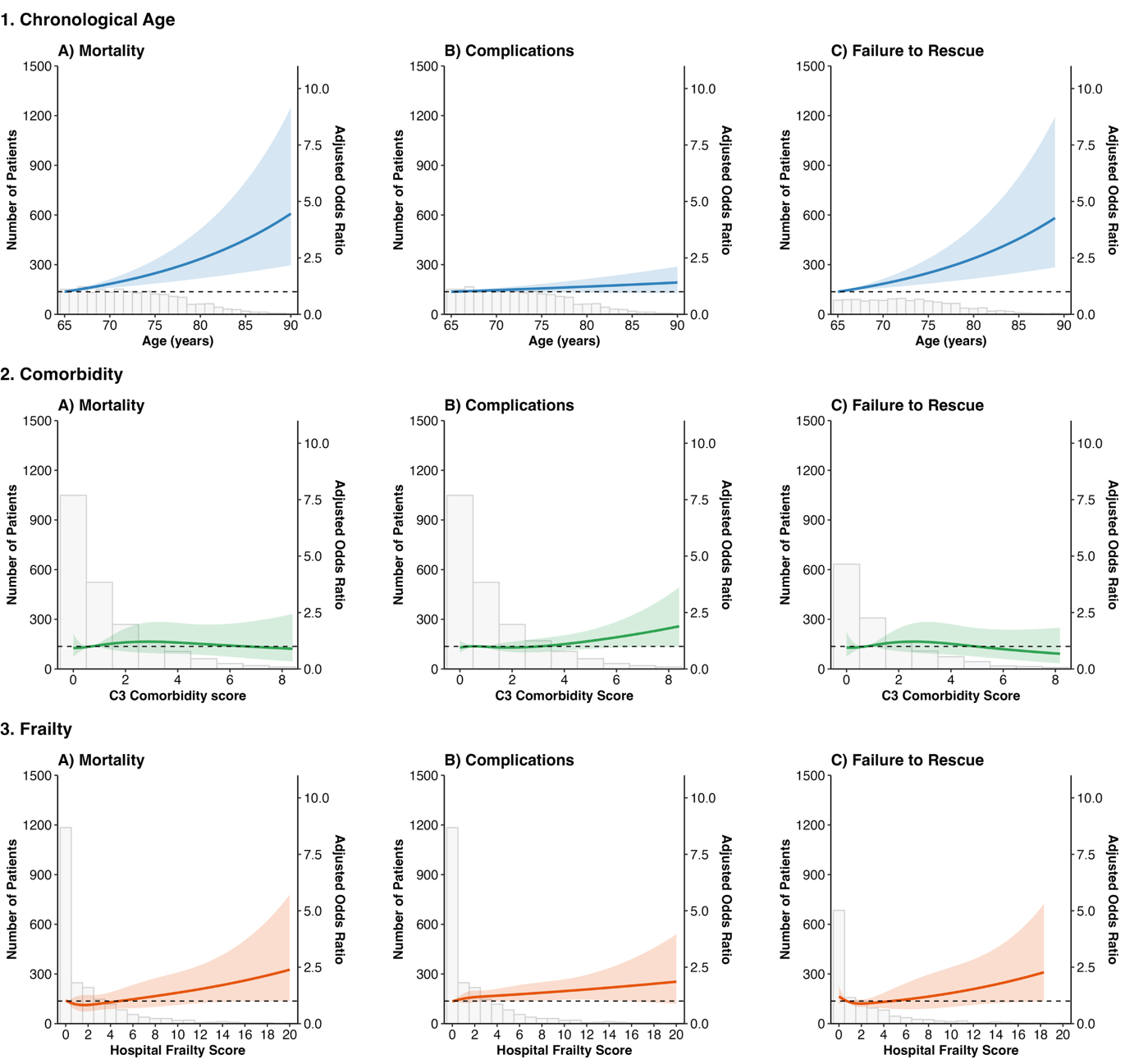


**Supplementary Figure 4**. Impact of chronological age, comorbidity, and frailty on mortality, complications, and failure to rescue amongst patients undergoing elective surgery for non-metastatic cancer.


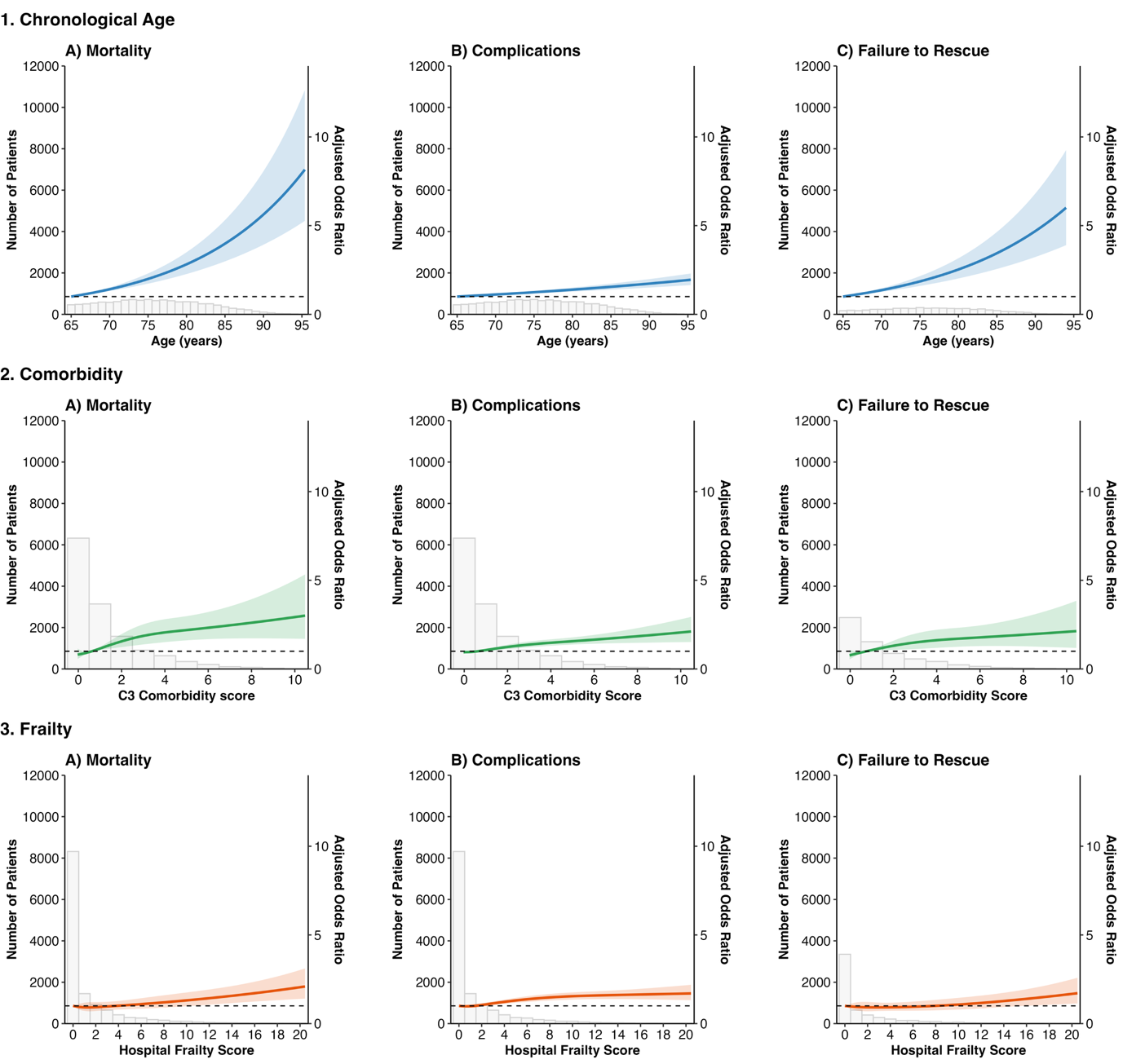

Supplement: Supplementary file 1 — Supporting Information S1 [file WJS-50-713-s001.docx]
